# Negotiating knowledge: The role of network hedging in the production of high-impact science

**DOI:** 10.1371/journal.pone.0352349

**Published:** 2026-06-29

**Authors:** Adrián A. Díaz-Faes, Pablo D’Este, Anne L. J. Ter Wal

**Affiliations:** 1 INGENIO, CSIC-Universitat Politècnica de València, València, Spain; 2 Imperial Business School, Imperial College London, South Kensington Campus, London, United Kingdom; Leiden University: Universiteit Leiden, NETHERLANDS, KINGDOM OF THE

## Abstract

Extant research shows that knowledge networks with a greater diversity of participants and richer in structural holes are more conducive to advancing science and innovation. However, research on network structure and network composition tends to overlook how actors might best utilize their connections. In this study, we seek to unpack the variation in how individuals leverage the opportunities afforded by their networks, focusing on the scientific knowledge production process. Given the uncertainty faced by scientists during the different stages of the research process, we argue that network hedging – consulting multiple individuals for the same resource need – rather than network compartmentalization – turning to a particular network contact for a specific resource need – increases the likelihood of research findings with greater scientific impact. By analyzing granular data on the network mobilization decisions and scientific outputs for a sample of biomedical scientists, we find support for our prediction that network hedging is positively associated with the production of high-impact scientific output, and that this effect is manifest beyond the benefits of being embedded in more diverse or sparser networks.

## Introduction

*“The ‘ideas’ of the laboratory are social occurrences which emerge from*

*interaction and negotiation with others”* [1] (p. 13).

Extant research shows that networks with more diverse participants and richer in structural holes are more conducive to advancing science and innovation. In line with Schumpeter’s notion that science and innovation stem from recombinatorial processes [2,3], it is argued that individuals whose networks span more varied groups or whose contacts are only loosely interconnected have a vision advantage to spot new opportunities for knowledge recombination over those with less diverse or overly closed networks [4,5]. However, we argue that the advantages that stem from network structure and network composition are only half the story [6,7]. The degree of diversity and presence of structural holes in a network shape individuals’ access to information and their opportunity space, yet such purely structural factors do not explicitly consider how individuals *utilize* their connections. Given that “networks do not act, [but] are a context for action” [4] (p. 354), the other half of the story includes the variation in how individuals leverage the opportunities afforded by their networks.

This variation might arise from decisions about whether to compartmentalize network contacts – i.e., to consult a particular contact for a specific resource need – or hedge them – i.e., collect and contrast inputs from multiple connections for the same need. Each individual is likely to approach these decisions differently. While compartmentalization reduces the burden on the individual’s network connections and is justified by wanting to access the person best able to help on a given matter, hedging elicits benefits from triangulation by allowing access to multiple views on the same problem or the same resource need.

Building on a growing stream of research on network activation and utilization decisions [8–11], this paper seeks to explore network mobilization decisions in the context of scientific knowledge production. Specifically, it explores how and to what extent scientists’ network mobilization affects their ability to generate high-impact scientific outputs. We argue that network hedging rather than network compartmentalization helps the scientist to produce greater scientific impact. Our argument rests on the claim that, given the uncertainty inherent in scientific work [12,13], the benefits of accessing multiple viewpoints on the same problem outweigh the potential costs of overburdening the scientist’s network.

Analyzing granular data on the network mobilization decisions and scientific outputs of a representative sample of Spain-based biomedical scientists, we find support for our prediction that network hedging rather than network compartmentalization is positively associated with the production of high-impact scientific output. We show also that this effect is manifest beyond the benefits of a more diverse or a sparser network. In documenting how network hedging outperforms network compartmentalization in overcoming the uncertainty inherent in the production of high-impact science, we delineate a critical and previously underexplored network decision made regularly by scientists when seeking to leverage their social capital for input and advice. That is, we unpack network behavioral mechanisms, frequently implied in network research but rarely measured empirically, to observe in granular detail how the practice of reaching out to multiple contacts for the same resource underpins network advantages in scientific research.

## Theory

### Uncertainty in the scientific research process

“If a research experiment were well defined, it would be unnecessary to perform it (…) the more unknowns there are and the newer a field of research is, the less well defined are the experiments” [14] (p. 86). Scientific knowledge production is an activity whose inherent uncertainty afflicts scientists throughout the different stages of the research process [15].

To begin with, there is uncertainty surrounding the scientist’s decision about which questions, ideas, or problems to focus on. What is new in a particular field and which questions are worth exploring are often contested. The merit and feasibility of ideas with the potential to advance scientific understanding are fundamentally unknowable [12,16], and this uncertainty is higher in the case of research that traverses truly uncharted territory. Without embarking on research to thoroughly explore and investigate new possibilities, it is impossible to decide whether their exploration is worthwhile. At the same time, high-risk projects are hard to justify precisely because their potential impact is inherently uncertain from the outset [17]. In the early 2000s, Katalin Kariko struggled to persuade science funders of the potential of mRNA molecules for human therapeutic application. Only after almost 20 years and many unsuccessful funding bids – and thanks to her conviction and perseverance – did their potential become clear when used to develop coronavirus vaccines [18].

Once scientists have settled on a given question, the most effective course of action (e.g., most appropriate research design or method) may not be obvious. Given time and budget constraints, scientists need to whittle down the number of possible methodological pathways in the absence of full awareness or complete knowledge of the alternatives. These difficulties, particularly salient when exploring new phenomena or adopting unproven or unconventional methodologies [14], are further compounded by the long-term implications of early courses of action. Owing to the path-dependent nature of scientific knowledge production [19] and cognitive constraints on scientists’ abilities to deviate from an initial course of action, early decisions about research approaches can have far-reaching and often unanticipated consequences [14].

The analysis and interpretation of scientific data also involve mitigating uncertainty. Whether research findings emerge on the basis of accumulated evidence or paradigm shifts, interpretative processes are “noisy” because the judgments and the heuristics that underpin them may vary from person to person and might be time- and place-contingent [20]. How different pieces of a puzzle fit together to form a coherent picture and narrative might, for example, depend on the sequence in which different elements of the evidence emerge; and the importance of certain findings, as perceived by scientists, will depend on the quality and nature of their previously accumulated scientific knowledge [12,21,22]. Research on Alzheimer’s disease for long focused on preventing its onset and progression but no specific measures have proven effective so far. Recent findings and the approval of two anti-amyloid antibodies as potential treatments offer a possible roadmap for slowing the disease [23]. However, the absence of a unified theory and limited understanding of the disease’s pathogenesis suggest that access to an effective therapy is a long way off [24].

Finally, scientists also face uncertainty when validating their research ideas and findings. Skepticism may apply to both decisions about the right questions to pose and the evidence and narrative as to why the questions and results are important. When philosopher William Hamilton took aim at phrenology – the “science” of judging a person’s mental traits through the measurement of bumps on the skull – he conducted experiments that contradicted phrenologist George Combe’s hypothesis that the cerebellum controlled sexual activity and was more prominent in men than women. Hamilton challenged Combe to find a single anatomist who would stake their reputation on the veracity of phrenology. Combe responded that phrenology was estimative and expressed his contempt for what he deemed biased expert opinions and instead relied on popular opinion to validate his theories. Phrenology lost and since then has had no place within the bounds of science [25].

### The role of network hedging in mitigating uncertainty and increasing scientific impact

It has long been established that advice and collaboration networks play a pivotal role in scientists’ efforts to achieve significant advances in science [26–28]. Scientific history offers an abundance of examples of collaborative and advisory relationships that have shaped the direction of travel in scientific enquiry. The term “invisible college” describes a group of geographically and institutionally distant scientists who regularly interact on the basis of a shared scientific interest [29,30]. The term was coined to describe a group of scientists in the mid-17^th^ century who later organized formally into the Royal Society of London. As Price and Beaver [31] (p. 1101) illustrate, this group of scientists “communicated by letter to gain an appreciative audience for their work, to secure priority, to get approbation from peers, and to keep informed of work being done elsewhere by others.”

Contemporary research on scientific networks – and on the role of networks in creativity, science, and innovation more broadly – focuses primarily on the role of network structure and network diversity in promoting science and innovation [32–37]. There is wide consensus that in terms of identifying “good ideas” individuals whose networks span structural holes – that is, individuals who broker otherwise disconnected individuals or groups – have a vision advantage [4]. For instance, there is ample evidence of brokerage being associated positively with creativity, scientific progress, and innovation [38–40]. Relatedly, it is often argued that individuals whose networks span domain boundaries, cut across communities, or draw on a broader range of knowledge areas have better access to diverse information [41–43] and outperform those with more homogeneous, cliquish networks [5].

Taken together, extant research into the structural and diversity benefits of networks has advanced our understanding of how knowledge networks shape the opportunity space for scientists to produce valuable ideas. However, it does not show how scientists’ day-to-day decisions about which of their network connections to consult might help mitigate the uncertainties integral to their scientific pursuit. To shed light on how scientists’ decisions about leveraging their network connections for input and advice matter for knowledge generation, we conceptualize the network mobilization decisions on a continuum going from network compartmentalization to network hedging. Network compartmentalization involves scientists having different “go-to” partners for each specific resource need. Compartmentalizing scientists reach out to the specific individual network member deemed best suited to a given resource need. Motivated by a desire to make the most efficient use of the network’s resources, compartmentalization involves precise judgment about who is best placed to help over a given problem or resource need.

In contrast, network hedging involves the scientist reaching out to multiple parties for the same resource need. Although hedging likely increases the burden the scientist places on each of their network contacts (especially if network size is fixed), we argue that network hedging is more effective than network compartmentalization for helping the scientist to mitigate the uncertainties associated with research activity and production of high-impact science outputs. Below, we describe how network hedging contributes to mitigate uncertainty in the production of high-impact scientific outputs. We adopt the three criteria of scientific merit (originality, plausibility, and scientific value) proposed by Polanyi [44] and add a fourth condition of societal relevance to respond to concerns about the broader socio-economic impact of research [45,46].

*Originality.* Network hedging allows scientists to build a more balanced view of which ideas or questions are most worth exploring. Early-stage science ideas that traverse uncharted territory or draw upon atypical knowledge combinations are likely to elicit highly divergent reactions [47,48], some highly dismissive and others more supportive and perhaps sharing the same excitement – see also [10,11]. Scientists who compartmentalize their networks risk a promising idea being abandoned should the single network contact they chose to consult be dismissive. At the same time, if the sole source of advice does not challenge misconceptions in relation to the first principles involved, the scientist might continue to explore dead ends for too long. In contrast, individuals adopting a network hedging approach will be more likely to receive a mix of reactions, which might allow them to better assess the originality of a research problem and also abandon seemingly futile research directions. The greater the variety of the viewpoints gathered, the more informed the scientist’s choices and decisions about research objectives and aspirations. This more balanced base enables abandonment of excessively risky projects and a focus on promising ideas, favoring the identification of the sweet spot of less risky and more original and potentially high-impact research.

*Plausibility.* Network hedging allows scientists to enquire about the soundness and solidity of the research. Given the path-dependent nature of scientific exploration and the strong cognitive constraints on scientists’ abilities to deviate from a given direction, it is imperative to consider a broad set of alternatives and to compare the pros and cons of a range of methods rather than making a premature decision based on the recommendation of a single contact. Network compartmentalization leaves scientists more open to confirmation bias. As a result of the principles of homophily [49], there is a higher likelihood that the scientist will turn to the network contact with the most similar views [50] and to selectively filter out from a single conversation those bits of information that validate their initial thinking [51]. On the contrary, network hedging enables scientists to gather multiple and complementary views which help them to navigate uncertainties about the potential merits, risks, and obstacles involved in different research methods. For instance, were the scientist to favor an unconventional method, network hedging would be effective to ensure that the research design is more consistent with professional science standards. New information that perhaps conflicts with the proposer’s own view, may be considered credible only if confirmed by multiple sources [52] – scientists are often reluctant to change their view on the basis of feedback from their closest ties [53]. Thus, access to multiple views through network hedging enables the scientist to construct a more comprehensive picture of the most appropriate approach and reduces the risk of adopting flawed designs which pluralistic input might have helped prevent.

*Scientific value*. Relatedly, network hedging also helps to increase analytic accuracy by allowing scientists to better determine the significance of their results and articulate the most coherent narrative. Given that analysis and interpretation are often not straightforward, by getting “the same story told different ways”, individuals can piece partial stories together into a single coherent one [54,55]. Shannon and Weaver’s [56] mathematical theory of communication posits that it is the redundancy of input that allows the individual to interpret noisy information signals derived from an understanding of different elements through different channels [22]. When working on challenging projects at the bleeding edge of science, it is imperative that scientists collate different interpretations of the same data, identify and interpret overlaps and differences, and on this basis make inferences about the appropriateness and accuracy of their own interpretations [57–59]. Furthermore, multiple informants allow individuals to test out different narratives and combine a range of elements into a story that is, overall, more compelling and more likely to appeal to the research community [55,60]. In the generation of scientific claims, network hedging helps to balance both “conviction with caution” [61] and also deepness with broad-mindedness [62], and enables experimentation with alternative framings [63]. Formulating a narrative about why research ideas and findings are important and ensuring that it is perceived by peers as portraying scientific value, is often the result of involvement in a highly distributed knowledge process. For instance, as Knorr-Cetina [1] points out, the final version of a scientific article is the product not just of its authors but also of other scientists whose critical comments have been taken into account – the outcome of an often lengthy process of sensitive compromises between authors and critics.

*Societal relevance.* In addition, network hedging also procures a more compelling vision of the significance of research findings beyond academic boundaries. Reaching out to different people for the same resource need is more likely to elicit a comprehensive view of the practical relevance of potentially ground-breaking research outside academia. Gathering multiple viewpoints contributes to judgements about which research directions will be more responsive to societal challenges and demands. Multiple views allow that even research which is mostly aimed at a scientific audience is able to communicate its claims [21] to a wider audience and emphasize the broader relevance of the contribution, beyond a particular scientific domain. Involving discussions with non‑academic actors may further expand the range of ideas and questions worth exploring, laying the groundwork for original and valuable scientific work.

Since originality, plausibility, value, and relevance parallel the dimensions on which conventionally the scientific merit of research work is assessed by the scientific community [44,46,64], we argue that scientists who engage in network hedging will be better able than scientists who compartmentalize their networks to mitigate the inherent uncertainties of the research process and increase the impact of their scientific work. More specifically, we contend that hedging represents a knowledge negotiation process which informs scientists’ decision-making in the context of scientific work and allows them to capitalize on the fruits of multiple inputs to mitigate uncertainty and enhance the scientific merit of the research. Ultimately, because scientists cite work they think has merit, the effect of hedging should be reflected in scientists’ ability to produce scientific outputs that are more likely to be highly cited. This effect will manifest beyond the influence of the structure and diversity of the network or the degree of uncertainty characterizing the research process. Accordingly, we hypothesize that:

***Hypothesis:*** *Network hedging (i.e., consulting multiple contacts in relation to the same resource need) is associated positively with the scientist’s ability to produce high-impact science.*

## Methods

### Context and data

The context of our study is the biomedical research community in Spain; specifically, scientists working for the Biomedical Research Networking Centers or CIBERs. The CIBER initiative was launched by the Spanish Government in 2007 to foster research excellence in biomedicine and support translational research to enhance the quality of healthcare service [65]. Pivotal to the CIBER program is enhancement of research cooperation among scientists working on similar pathologies in different research labs and institutional settings (i.e., universities, hospitals, public research organizations, industry, etc.). Open calls to join the CIBER program focused on a specific range of pathologies and diseases represented by multiple biomedical specialties. Participants are selected based on responses to highly competitive calls. The present research project was supported and approved by CIBER’s scientific directors.

We consulted public records and annual reports to collect contact information for members of the research labs in the CIBER program. At the time of the study, this population comprised 4,758 biomedical research center staff with varying levels of seniority (i.e., principal investigators, early-stage researchers, senior researchers) along with technicians and management support staff. In the first stage of our study, we conducted 16 interviews with several CIBER scientific directors and research lab principal investigators. These interviews provided us with a contextual understanding of the types of resources biomedical scientists require for their work and how they use their interpersonal connections to obtain them. In the second stage, we conducted a survey administered in April 2013 to the entire population of CIBER scientists. Prior to participation, informed written consent was obtained from all respondents. Participants were informed about the purpose of the questionnaire and were given details on how their personal data and responses would be treated. Responses were anonymized and assigned an alphanumeric code. The survey was designed to capture the networks on which biomedical scientists rely in their activities to generate new scientific knowledge, as well as attitudes and sociodemographic variables. We received 1,309 responses, a response rate of 27.5% which is similar to other surveys of academic scientists – cf. [66,67].

To assess the effect of networks on scientific impact, we matched survey responses to publications (citable items: articles and reviews) from the Web of Science (WoS) database. Using the Leiden University Centre for Science and Technology Studies (CWTS) in-house database which disambiguates author names in raw WoS data, based on an unsupervised disambiguation method [68], we were able to match 1,013 (77%) of our survey respondents to a WoS publication profile. We captured publication data covering the periods before (2000–2012) and after (2013–2017) the survey which is the source of our dependent variable and several control variables. Technicians and management support staff, and a small number of scientists not actively publishing during the period examined, were removed from the study. The number of usable responses was further reduced due to missing responses to some of the questions relevant for the study. The resulting sample of 771 biomedical scientists is representative of the full population of 4,758 CIBER scientists in terms of the distribution of responses across biomedical specialties (see S1 Section). We conducted several tests for non-response bias and also classified the sample into early and late respondents based on the date that the questionnaire was returned. We performed a wave analysis to check whether early and late responses differed in terms of lab size, network size, and the total number of publications in the period 2013–2017. Late respondents were used to proxy for the response patterns of non-respondents [69]. Differences in means across the three variables were all rejected in statistical terms.

### Measures

#### Dependent variable.

This study draws on highly cited publications as an indicator of scientific impact. We argue that citation count (appropriately normalized) captures the four characteristics of scientific merit discussed in our theorizing.

Scientists cite prior work to give credit to previous research, identify methodologies, substantiate claims, and provide background reading, but citing behavior can be also influenced by factors beyond the merits of the focal publication – for example, language biases or target audience [46]. However, it is generally agreed that citations measure scientific impact and that highly cited publications usually represent significant scientific research [70,71]. Therefore, we assume that *original* research will be more frequently cited [48,72] although certain highly original contributions suffer delayed recognition [47,73]. Citation patterns can also reflect *plausibility* and methodological soundness to which the journal peer-review system contributes [74]. Similarly, it is reasonable to assume that highly cited publications carry greater *scientific value* than those that are never or rarely cited. For instance, it is well-known that citations correlate with honorific awards, prizes, and academic promotion [75] and usually reflect “actual influence (impact) on surrounding research activities at a given time” [64] (p. 70). Last, although citations account primarily for scientific use and therefore are not sufficient to assess *societal relevance*, it has been shown that highly cited publications tend to have greater societal relevance measured by the capacity to attract the attention of non-academic audiences in news media, social media, or policy documents [46,76]. For these reasons, and despite their limitations, citations provide valuable insights for assessment of scientific impact.

Thus, to measure the scientist’s ability to generate high-impact research findings we use the number of highly cited publications – that is, number of publications published by the scientist in the five years following our survey (2013–2017) that are among the top 10% most frequently cited among publications published in the same field and the same year: *High scientific impact (P*_*top10%*_*)*. Normalization by field and publication year allows for better comparison across different scientific domains and/or time periods [77], as this allows us to rule out the effect of different citations practices and publication age. Given the high right-skewness of the distribution of citations, a count is more appropriate than an average citation measure. As Seglen [78] shows, skewness is a distinctive feature of citations data: given any set of publications there will always be a large fraction of uncited publications and a small fraction which accounts for most of the citations in a given field.

#### Independent variables.

We measured the extent to which scientists engage in network hedging, and the structure and diversity of their personal networks, focusing on connections outside the scientist’s own research lab. In our survey, we adopted an ego-centric approach using name-generator questions with free recall [79]. That is, we instructed respondents (*egos*) to name up to 10 contacts (*alters*) outside their own research lab who were “particularly important for the advancement of their research activities during the year 2012”; thus, values for the size of an individual’s network range from 0 to 10. S2 Section contains full details of the network questions included in the survey.

*Network hedging*. To measure scientists’ decisions about the network connections to mobilize for a resource need, we asked them to indicate the precise resources obtained from each contact. We based the list of resources on our initial interviews, adapting the scale developed by Cross and Sproull [80] and later validated by Levin et al. [81] and Walter et al. [82] to capture a broad range of resources accessed from interpersonal connections for the advancement of scientists’ research activities. The list of resources included the following items: help to define or reframe a problem (*problem reformulation*); help with specific solutions to problems or technical advice (*problem-solving*); suggestions about other sources of information (people, archives, databases) not previously considered (*referral*); validation of plans and solutions and bolstering confidence (*validation*); legitimation of ideas based on support from influential peers (*credibility*). These five resource needs broadly map onto the four challenges discussed in the Theory section: input to problem reformulation provides the scientist with the necessary resources to assess the research direction; inputs to problem-solving and referral enable the scientists to identify the appropriate design and methodology; inputs aimed at validation and credibility help the scientist to correctly interpret the findings and appropriately frame the narrative in terms of scientific value and societal relevance.

**Fig 1:**
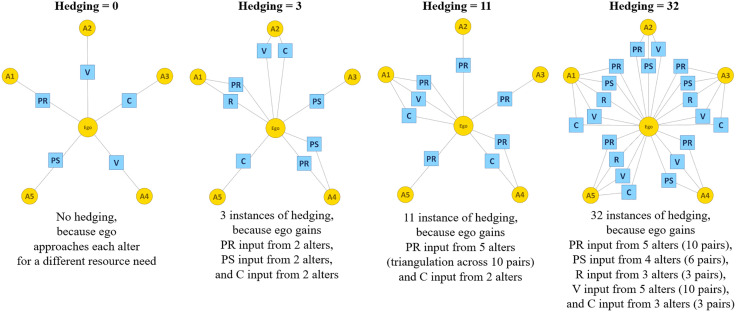
Network hedging. *Notes:* Nodes A1 to A5 represent five alters in ego’s network. PR = Problem Reformulation; PS = Problem Solving, R = Referral; V = Validation; C = Credibility.

We measure network hedging as the total number of times the scientist triangulates across contacts for the same type of resource, defined as follows: $\sum_{i=\text{1}}^{\text{5}}T_{i}$, where $T_{i}$ denotes the number of unique alter pairs mobilized for each resource need *i*. As depicted in Fig 1, the more frequently the scientist reaches out to multiple network contacts for a given resource need, the higher the triangulation count, and hence, the higher the hedging measure. The less frequently the scientist relies on multiple contacts for the same resource – that is, the greater the degree of network compartmentalization – the lower the hedging measure. Note that we aggregated our triangulation count across all resources. This allowed us to use a single hedging measure for each respondent, although some resource needs may be more relevant to some respondents than to others. Although an overall count is our preferred specification due to complications associated with ratio-based variables [83], in a robustness check, we also computed the average number of alters mobilized per resource need (see Results section).

*Network brokerage.* To capture the network structure, the survey asked respondents for information on each pair of reported alters [84,85]. This allowed us to trace each respondent’s ego network structure. We computed network brokerage by counting the number of structural holes in each personal network [86], that is, the absence of alter–alter ties within a personal network. This number was divided by the total number of possible alter–alter ties: [*n* (*n* – 1)/ 2]. The network brokerage measure ranges from 0 to 1, with low values reflecting a network with few structural holes and high values reflecting a network rich in structural holes.

*Network diversity*. Research shows that personal networks that cut across distinct professional domains are an important source of network diversity [40,41,87]. To measure network diversity, we asked respondents to classify their listed alters into one of the following professional communities: basic scientist, clinical scientist, medical practitioner, patient or patient representative, private industry scientist, public administration employee, and other. We obtained a measure of diversity rather than concentration, by calculating the Herfindahl index and subtracting it from 1. Hence, our network diversity measure is calculated as follows: $\text{1}-\sum_{i=\text{1}}^{c}(n_{i}/\;N)$^2^, where *c* is the number of professional communities, *n*_*i*_ is the number of contacts belonging to the professional community *i,* and *N* is the total number of contacts. Values range from 0 to 1: values closer to 0 indicate a homogeneous network; higher values reflect a more diverse one.

#### Control variables.

We included a range of control variables. First, we included a past scientific impact variable (*Past scientific impact*: *PP*_*top10%*_ *2000–2012*); this measure accounts for the proportion of the respondent’s publications in the period 2000–2012 which, when compared with other publications in the same field and in the same year, were among the top 10% most frequently cited. We also included a count of prior publications (*Total pub 2000–2012*). Including these past performance measures can be used to approximate the role of unobserved individual attributes which might underpin the scientist’s ability to produce high-impact scientific outputs in both the past and the focal analysis period.

Second, to assess the uncertainty related to the research process, we computed a measure estimating the degree to which the scientist conducts research that involves crossing cognitively distant disciplinary boundaries (*Cognitive disparity*). We relied on the reference lists of publications published in the period 2013−17 and computed the average cognitive distance between the WoS subject categories involved. Each journal in the WoS database is classified into up to six subject categories from a total of 254. Our measure of *Cognitive disparity* is based on the distribution of journal subject categories cited in the reference list in a given publication. We followed a similar procedure to Yegros-Yegros et al. [88] and created a matrix of co-citation flows between WoS subject categories which then was converted into a Salton’s cosine similarity matrix. The cognitive distance between two subject categories is computed as *d*_*ij*_ = (1 – *s*_*ij*_), where *s*_*ij*_ is the cosine similarity between each pair of subject categories *i* and *j*. We obtained a mean disparity value for each publication and calculated the average across publications to obtain a measure at the individual scientist level. Values range from 0 to 1, with higher values corresponding to more cognitively distant research. While recognizing that cognitive disparity is not necessarily equated to uncertainty, recombining knowledge from distant fields usually involves greater research uncertainty than conducting research within the confines of a single discipline [72]. Thus, research involving atypical combinations of knowledge is likely to involve more uncertainty [47]. Since prior research suggests diminishing returns [47,88] to combining disparate knowledge in terms of achieving high scientific impact, we expect an inverted U-shaped relationship between the two. Thus, we use a linear and a quadratic term for cognitive disparity.

Third, we account for gender and seniority by including dummy variables for whether the scientist is *Female* and whether the scientist is a *Principal investigator*. To account for scientists’ job characteristics, we included dummy variables for CIBER’s nine areas of biomedical specialty (ref. category: *bioengineering, biomaterials and nanomedicine*) and type of institution (*university*, *hospital*, *public research organization*, or *other –* ref. category) employing the scientist. To further characterize the scientist’s research focus, we employed a survey-based measure to indicate research with a *Basic orientation* as opposed to being applied research, and included categorical variables for the proportion of work time dedicated to different activities (*research time*, *teaching time*, *contact with patients*, *administrative duties time*, *building professional links*, or *other activities –* ref. category).

Fourth, we account for skill and personality including a measure for *Breadth of skills*, operationalized as the number of specialty areas in which the respondent was trained, plus five continuous variables derived from the survey responses to measure the Big Five personality traits of *Conscientiousness*, *Neuroticism*, *Openness*, *Extraversion*, and *Agreeableness*. Finally, we included variables for the individual’s local and international networks, including a count of the number of members of their research lab (*Lab size*), number of contacts important for the advancement of their research activities within their research lab measured by a separate name-generator question in the survey (*Lab contacts*), number of relevant contacts outside of the scientist’s research lab (*Network size*), and proportion of international collaborators (*PP*_*international collab.*_) measured as the proportion of publications resulting from international scientific cooperation (i.e., co-authorship) published in the five years following the survey (2013–2017).

## Results

### Descriptive statistics and hedging patterns

Table 1 presents the average values for the number of contacts the biomedical scientists consulted for a particular resource need, and the number of times these resources are triangulated. On average, scientists more often turn to multiple contacts for input related to thinking through a problem (x̄ _problem reformulation_ = 2.60) and solving a particular problem or technical issue (x̄_problem solving_ = 2.56) but tend to rely more on specific contacts to legitimate their research (x̄_validation_ = 1.42, x̄_credibility_ = 1.71). Our hedging measure reveals some interesting nuances. Problem solving is the most often triangulated resource need (x̄ = 5.95), followed by inquiring about relevant sources of information (x̄_referral_ = 4.31). Consulting colleagues perceived as experts is the least frequently hedged resource need (x̄_credibility_ = 1.98).

**Table 1:** Average values for resource needs in network mobilization.

	Nº of contacts			Instances of hedging		
Resource need	Mean (Std. Dev.)	Min	Max	Mean (Std. Dev.)	Min	Max
Problem reformulation	2.60 (2.28)	0	10	3.12 (6.69)	0	45
Problem solving	2.56 (2.24)	0	10	5.95 (9.38)	0	45
Referral	2.38 (2.19)	0	10	4.31 (8.29)	0	45
Validation	1.42 (1.89)	0	10	2.37 (5.79)	0	45
Credibility	1.71 (2.09)	0	10	1.98 (5.43)	0	45

**Fig 2:**
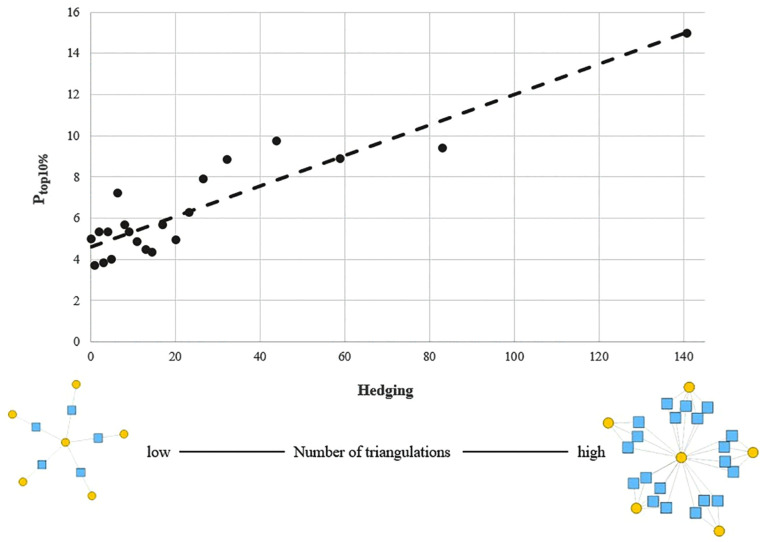
The association between scientists’ network hedging and the number of high-impact publications. *Notes*: Graph points depict average network hedging values for 30 groups of observations with increasing levels of network hedging.

Table 2 provides the descriptive statistics for all the variables and S3 Section plots their correlations. The scatterplot in Fig 2 shows scientific impact increasing with increased network hedging (*r* = 0.23, *p* < 0.001). To simplify the display, scientists were classified into 30 observational groups on the basis of average network hedging values; the vertical axis representing number of highly cited publications (*P*_*top10%*_) for the period 2013–2017 and the horizontal axis representing mean level of network hedging from survey data corresponding to year 2012.

**Table 2:** Descriptive statistics (N = 771).

Variable	Mean	Std Dev.	Median	Min	Max
High scientific impact (P_top10%_)	6.01	9.29	3.00	0.00	89.00
Hedging	17.60	30.47	6.00	0.00	225.00
Network brokerage	0.51	0.39	0.67	0.00	1.00
Network diversity	0.23	0.25	0.18	0.00	1.00
Cognitive disparity	0.47	0.08	0.46	0.12	0.75
Total pub 2000–2012	26.13	35.78	14.00	0.00	361.00
PP_top10%_ 2000–2012	57.52	31.07	64.58	0.00	100.00
Lab size	17.98	10.58	16.00	2.00	79.00
Lab contacts	5.31	3.34	5.00	0.00	26.00
Network size	3.94	2.86	4.00	0.00	10.00
PP_international collab._	38.40	30.09	33.33	0.00	100.00
Basic orientation	1.52	0.50	2.00	1.00	2.00
Breadth of skills	2.97	1.79	3.00	0.00	9.00
Conscientiousness	5.55	0.97	5.75	2.25	7.00
Neuroticism	3.35	1.07	3.25	1.00	7.00
Openness	5.41	0.98	5.50	1.75	7.00
Extraversion	3.97	1.16	4.00	1.00	7.00
Agreeableness	5.71	0.88	5.75	2.25	7.00
Female	0.50	0.50	1.00	0.00	1.00
Principal investigator	0.13	0.34	0.00	0.00	1.00
University	0.35	0.48	0.00	0.00	1.00
Hospital	0.23	0.42	0.00	0.00	1.00
Public research org.	0.10	0.30	0.00	0.00	1.00
Research time	55.85	27.73	56.00	0.00	100.00
Teaching time	11.25	14.36	7.00	0.00	90.00
Contact w/ patients	11.27	21.69	0.00	0.00	90.00
Admin. duties time	13.80	14.61	10.00	0.00	100.00
Building prof. links	5.59	5.91	5.00	0.00	30.00

### The relationship between network hedging and high-impact science

Table 3 presents the results of our main regressions. Since our dependent variable (number of highly cited publications) takes integer values and displays a highly asymmetric distribution, we employed negative binomial regression estimations. We rejected a Poisson model on the basis of the significant degree of overdispersion (deviance goodness-of-fit = 3152.756, *p* < 0.000). The baseline model (M0) shows that scientists with a strong track record of past (high-impact) publications, more international collaborators, and larger network size tend to generate more high-impact publications, while more oriented to basic research and those employed by a university tend to produce fewer high-impact publications. Moreover, we found that research involving knowledge from cognitively distant disciplines (i.e., cognitive disparity) displays a curvilinear relationship with number of highly cited publications.

**Table 3:** Results for negative binomial regression. Dependent variable: *P*_*top10%*_ (N = 771).

	M0	M1	M2	M3	M4
Hedging				0.046^***^ (0.011)	0.063^***^ (0.021)
Network diversity			0.071^***^ (0.017)		0.069^***^ (0.026)
Network brokerage		0.039 (0.053)			0.036 (0.063)
Cognitive disparity	0.071^**^ (0.036)	0.069^**^ (0.032)	0.069^*^ (0.039)	0.071^*^ (0.037)	0.067^*^ (0.038)
Cognitive disparity sq	−0.119^***^ (0.014)	−0.120^***^ (0.013)	−0.122^***^ (0.014)	−0.119^***^ (0.015)	−0.123^***^ (0.013)
Total pub 2000–2012	0.537^***^ (0.040)	0.535^***^ (0.041)	0.535^***^ (0.045)	0.537^***^ (0.041)	0.533^***^ (0.046)
PP_top 10%_ 2000–2012	0.444^***^ (0.087)	0.443^***^ (0.087)	0.446^***^ (0.088)	0.446^***^ (0.087)	0.448^***^ (0.088)
Lab size	−0.006 (0.028)	−0.005 (0.026)	−0.002 (0.026)	−0.005 (0.030)	0.000 (0.027)
Lab contacts	0.022 (0.039)	0.022 (0.039)	0.021 (0.043)	0.017 (0.039)	0.015 (0.041)
Network size	0.115^**^ (0.051)	0.099^**^ (0.041)	0.088^*^ (0.053)	0.080 (0.058)	0.025 (0.052)
PP_international collab._	0.237^***^ (0.032)	0.234^***^ (0.031)	0.238^***^ (0.031)	0.236^***^ (0.032)	0.234^***^ (0.030)
Basic orientation	−0.213^***^ (0.073)	−0.229^***^ (0.056)	−0.206^***^ (0.068)	−0.198^***^ (0.072)	−0.199^***^ (0.051)
Breadth of skills	0.036 (0.026)	0.034 (0.025)	0.033 (0.027)	0.039 (0.029)	0.035 (0.029)
Conscientiousness	0.033 (0.057)	0.033 (0.056)	0.033 (0.058)	0.032 (0.057)	0.033 (0.058)
Neuroticism	−0.041^***^ (0.013)	−0.040^***^ (0.014)	−0.038^**^ (0.016)	−0.041^***^ (0.012)	−0.037^**^ (0.015)
Openness	−0.020 (0.028)	−0.022 (0.030)	−0.025 (0.029)	−0.018 (0.027)	−0.025 (0.029)
Extraversion	0.001 (0.014)	0.000 (0.013)	−0.002 (0.013)	0.001 (0.015)	−0.003 (0.014)
Agreeableness	−0.007 (0.020)	−0.005 (0.022)	−0.009 (0.019)	−0.007 (0.019)	−0.007 (0.02)
Female	−0.091 (0.070)	−0.084 (0.065)	−0.092 (0.061)	−0.087 (0.069)	−0.080 (0.053)
Principal investigator	0.106^*^ (0.056)	0.115^*^ (0.067)	0.099^*^ (0.057)	0.096^*^ (0.054)	0.094 (0.064)
University	−0.077^***^ (0.020)	−0.075^***^ (0.018)	−0.092^***^ (0.021)	−0.080^***^ (0.019)	−0.093^***^ (0.022)
Hospital	−0.051 (0.107)	−0.055 (0.102)	−0.072 (0.100)	−0.061 (0.106)	−0.087 (0.095)
Public research org.	0.072^*^ (0.040)	0.073^*^ (0.042)	0.060 (0.039)	0.063 (0.040)	0.049 (0.040)
Research time	−0.026 (0.049)	−0.023 (0.045)	−0.017 (0.049)	−0.037 (0.052)	−0.030 (0.052)
Teaching time	−0.027 (0.042)	−0.031 (0.044)	−0.029 (0.041)	−0.032 (0.042)	−0.038 (0.044)
Contact w/ patients	0.024 (0.080)	0.024 (0.078)	0.028 (0.076)	0.016 (0.084)	0.018 (0.081)
Admin. Duties time	−0.034 (0.033)	−0.035 (0.033)	−0.037 (0.031)	−0.038 (0.032)	−0.043 (0.029)
Building prof. links	−0.007 (0.045)	−0.010 (0.046)	−0.010 (0.041)	−0.008 (0.045)	−0.012 (0.042)
CIBER dummies	Yes	Yes	Yes	Yes	Yes
Constant	1.514^***^ (0.083)	1.516^***^ (0.079)	1.521^***^ (0.078)	1.510^***^ (0.082)	1.518^***^ (0.073)
Cox & Snell *R*^2^	0.570	0.571	0.572	0.571	0.574

*Notes*: Robust standard errors (in parentheses) are clustered by respondent’s type of institutional affiliation.

*** p < 0.01, ** p < 0.05, * p < 0.10.

Model 1 (M1) adds our network brokerage variable, and Model 2 adds the network diversity variable. While we find no statistically significant association between network brokerage (*β* = 0.039, *p* = 0.458) and scientists’ ability to generate high-impact science outputs, it seems that scientists with more diverse networks (*β* = 0.071, *p* = 0.000) tend to produce more high-impact publications. A one standard-deviation increase in network diversity from the mean increases the predicted number of top publications by 0.31 points. Model 3 adds network hedging and model 4 is the full model. We find support for our prediction that greater network hedging is associated positively with a scientist’s number of high-impact publications (*β* = 0.063, *p* = 0.003). Based on the full model (M4), a one standard-deviation increase in hedging from the mean increases the number of high-impact publications by 0.27 points.

### Robustness checks

We conducted several robustness checks. First, we replaced network size and our network brokerage measure with network constraint [84] but found no statistically significant association (*β* = 0.038, p = 0.483) with the number of high-impact publications. Second, we tested the effect of network hedging behavior using an alternative measure – average number of alters per resource need. S4 Section shows that this alternative measure of hedging, which is strongly correlated with our main measure (*r* = 0.90), is also positively and significantly (*β* = 0.089, *p* = 0.017) associated with the number of high-impact publications. Third, we computed an alternative indicator for high scientific impact using a dummy variable which takes the value 1 if the biomedical scientist published seven or more highly cited publications (75^th^ percentile) and 0 otherwise. A quarter (25%) of the scientists had authored seven or more highly cited publications, which means that the score 7 corresponds to the P75 for the distribution of our measure of scientific impact. The results of the logistic regression are consistent with our main analysis (see S5 Section). Fourth, we reran our analyses excluding individuals who mentioned only one or two external network contacts, which meant limited opportunities for hedging. S6 Section provides robust support for the effect of network hedging (*β* = 0.057, *p* = 0.019) for this reduced sample (*n* = 505). Fifth, given the right-skewed distribution of hedging values we checked for the potential effect of outliers. In S7 Section we performed winsorization on the top 5% and 10% of observations and still found robust support for our hypothesis.

Sixth, it could be argued that the number of triangulations is an endogenous measure, and thus, the partial effect observed for hedging could be driven by unobserved factors. To explore the extent of any endogeneity concerns, we checked whether the geographical proximity of the scientist’s network contacts could be driving the individual’s opportunities to hedge. Geographical proximity favors social interaction and trust building with local actors [89], particularly in research networks that include academic and non-academic actors [90]. In this case, we would expect scientists working in research labs located in Spain’s major cities to have more opportunities for hedging than those in “peripheral” locations. We ran a U-Mann Whitney test for our sample of 771 biomedical scientists (by comparing 476 scientists located in Madrid or Barcelona and 295 scientists located elsewhere) but found no evidence of statistically different hedging medians (S8 Section).

Seventh, due to the cross-sectional nature of our data, our results could be affected by reverse causality. It might be that (past) scientific performance determines network hedging behavior rather than vice versa. To explore this, we performed a Granger [91] causality test. We exploited information from a second survey wave administered in 2018 which asked biomedical scientists participating in the CIBER program about the five types of resources used to compute our hedging measure (network data refer to 2016−2017). We conducted this analysis on the 202 biomedical scientists who responded to both survey waves. If the causal relationship between high scientific impact and network hedging went in the opposite direction to that hypothesized in our study, past performance should be a significant predictor of hedging. However, as shown in S9 Section, we found that the coefficient of past high scientific impact – *P*_*top 10%*_ – is not statistically significant (*β* = −0.029, *p* = 0.237). Instead, past hedging – *network hedging*_*t-1*_ – predicts future hedging – *network hedging*_*t-2*_ – (*β* = 0.377, *p* = 0.017), which suggests hedging behavior may be due more to personal behavioral preferences than a function of prior individual performance.

Finally, we considered omitted variables as a third source of endogeneity [92]. We followed the approaches of Frank [93] and Hill et al. [94] and computed the ITCV index for a non-linear model which estimates the extent to which a confounding variable must be correlated with the dependent and independent variables to alter our findings. To invalidate the effect of hedging on the scientist’s ability to produce high-impact outputs, 47.6% of estimates (367 cases) would have to be due to bias. This high percentage suggests that an omitted variable bias is not a serious concern.

### Boundary conditions

So far, we have shown that network hedging favors the production of knowledge with high scientific impact and provided additional empirical evidence to probe the robustness of our claim. However, it might be that the relationship between network hedging and high-impact science is contingent on specific features related to the research process. To test for potential boundary conditions on the examined relationship, we conducted the following analyses.

First, we checked whether high levels of hedging might have a detrimental effect on high scientific impact due to overburdening of the scientist’s personal network. We included in the analysis a quadratic term for hedging to test for evidence of a curvilinear (inverted U-shaped) relationship between network hedging and high scientific impact. We found no evidence of such a relationship (S10 Section). Second, we considered the potential interplay between network hedging and network diversity since greater heterogeneity in the composition of the personal network might provide more opportunities to leverage based on the triangulation of diverse perspectives. We found no statistically significant interaction between these two effects (S11 Section), which suggests different mechanisms at work for the influence of network hedging on scientific impact in a high heterogeneity vs. high homogeneity network.

Finally, we considered that the benefits derived from triangulating multiple viewpoints might depend on the degree of cognitive disparity in the scientist’s research work. As argued in the Theory section, network hedging is likely to be particularly beneficial in research contexts involving higher levels of uncertainty [95], such as research projects that require crossing cognitive boundaries and combining weakly connected research fields (i.e., high cognitive disparity). In this case, by drawing on plural expert advice to ensure more robust and rigorous judgements at every critical phase in the research process, network hedging might represent a behavioral strategy to inform decision-making in each phase of research.

**Fig 3:**
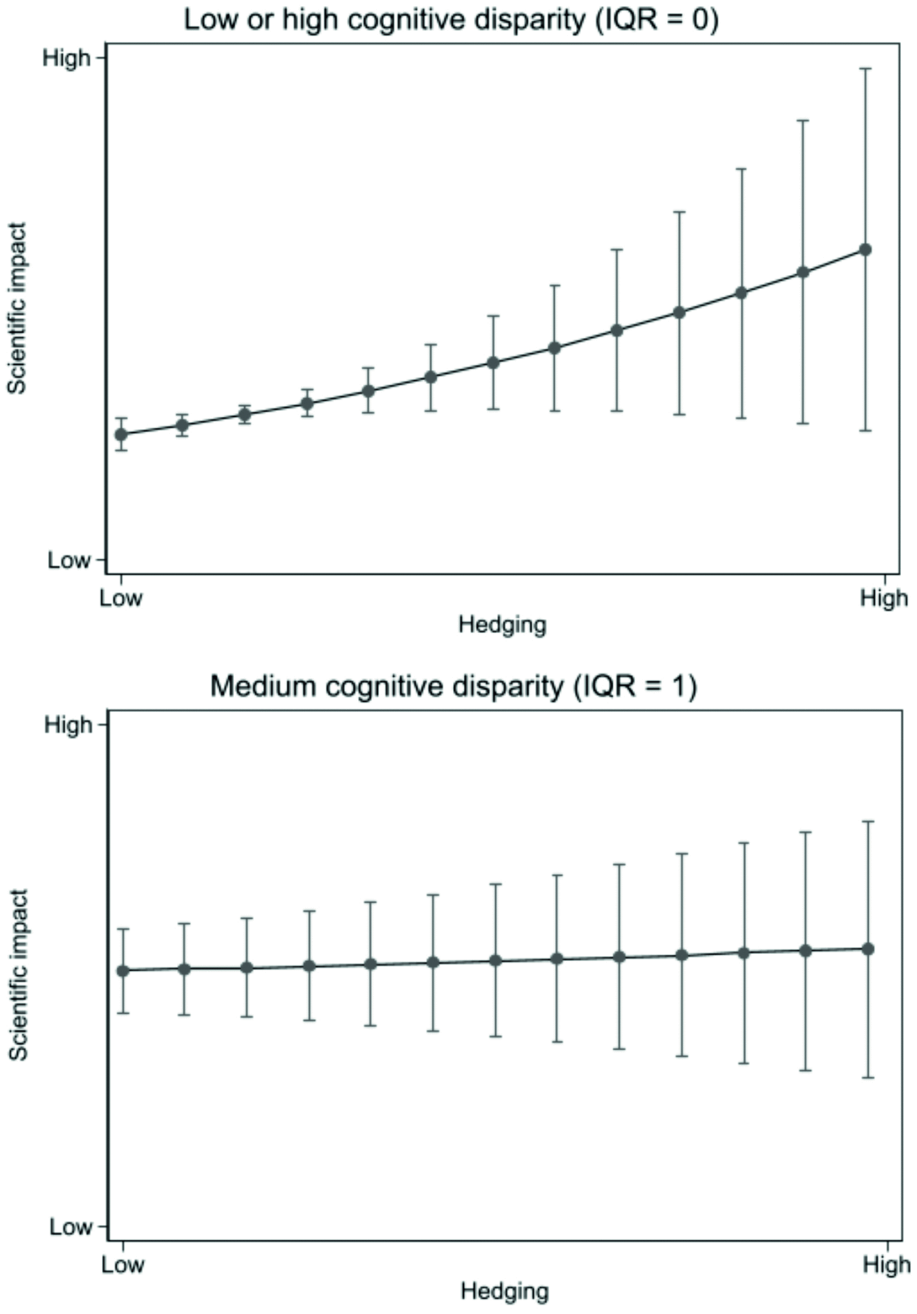
The relationship between network hedging and high scientific impact, for different levels of cognitive disparity. *Notes:* Predictive marginal effects of hedging with 90% confidence intervals, based on the split-sample analysis reported in Supplemental Section 10. The y-axis reports the predicted expected number of highly cited papers. IQR = 0 identifies cognitive disparity scores below the 25th percentile or above the 75th percentile; IQR = 1 otherwise.

Similarly, uncertainty might be associated with the unknowns and unpredictability in research projects with a strong focus on a particular disciplinary domain or projects that involve research in highly interconnected research fields (i.e., low cognitive disparity). In this case, network hedging might act as a behavioral mechanism to mitigate cognitive lock-in. By considering diverse perspectives and leveraging the opportunities afforded by their networks, scientists reinforce the capacity to delve deeper and more effectively into the original research goals within the confines of a particular discipline, while avoiding disciplinary-embeddedness and potential information redundancies. We employed a split sample procedure to test for this effect by computing a dummy variable based on the interquartile range (IQR) of *Cognitive disparity* (*IQR = 1 if P*_*25*_ *≤ cognitive disparity ≤ P*_*75*_*; 0 otherwise*), which covers the intermediate cognitive disparity scores. S12 Section shows that we found evidence of a positive and statistically significant association between network hedging and high-impact science in research processes that involve high and low but not medium levels of cognitive disparity. Fig 3 plots the predicted marginal effects.

## Discussion and implications

This paper explored how network mobilization decisions offer an additional layer of explanation about how networks facilitate the production of original ideas and creative outputs beyond the widely established effects of network structure and diversity. We showed that biomedical scientists who engage in higher levels of network hedging (i.e., reach out to multiple people for the same resource need) tend to produce more highly cited publications than scientists who compartmentalize their networks (i.e., consult a particular person for a specific resource need). This finding is important because it reinforces the view that network structure and network behavior can be complementary: while network structures are critical “contexts for action” which shape the opportunity space individuals can leverage to access information and other resources as key inputs for their work [4] (p. 354), network hedging reveals a particular type of action within those contexts which might explain additional variation in performance outputs. From this perspective, our paper makes two broad contributions.

First, building on a growing stream of research on network mobilization and utilization decisions [8–11], we addressed a critical dilemma faced by individual scientists seeking to leverage their social networks for input and advice. While network compartmentalization requires careful judgments about who is best placed to help with what, and avoids overburdening of the individual’s network with excessive requests for help, it comes with a critical opportunity cost. That is, unlike network hedging, it does not provide access to multiple, potentially divergent inputs and views on the same resource need. Although the compartmentalization–hedging trade-off may play out differently in other contexts, in scientific knowledge production the value of hedging outweighs the costs of overburdening the individual’s network, because triangulation is key to overcoming the uncertainty inherent in core scientific choices in relation to the direction, design, interpretation, and validation of research.

Second, we disclosed network behavioral mechanisms. These mechanisms are often implied in network research but rarely measured empirically, except in terms of separation (*tertius gaudens*) and cooperation (*tertius iungens*) brokering strategies [6,7,39,96,97]. Access to diverse inputs and viewpoints is at the heart of the prevalent arguments in our field about how networks enable pursuit of new ideas in science and innovation [4,38,98]. It has become commonplace to invoke precise mechanisms of how network structure and diversity enable or constrain access to core information, interpretative processes, and collective sensemaking [22,52,58,59]. In this study, we contribute to this body of work by observing more granularly how individuals utilize multiple connections for a particular resource need. Our approach brings to the foreground both the specific types of resources that flow through social ties and how a particular network mobilization and utilization decision helps the scientist to mitigate uncertainty and produce high-impact outputs by reaching out to multiple contacts for the same resource need.

Our study is not without limitations. Although we ran several robustness checks to address endogeneity issues, we are cautious about making causal inferences since the cross-sectional nature of our empirical design is intrinsically limited in terms of disentangling processes that might simultaneously influence the individual’s networking behaviors and performance outcomes. We encourage future research using longitudinal data and experimental designs to infer causality with greater precision. Also, our data refer to the context of biomedical research in one particular country: further empirical work could check whether our findings are robust to different contexts, scientific fields, and countries. Since our study does not decompose the benefits of hedging in terms of the different aspects of scientific work (i.e., research direction, research design, interpretation, and validation) future research could unpack how network hedging might help or perhaps hinder specific research tasks. Finally, future research could examine more directly the social capital costs implicit in network hedging, which we have only partially addressed at in this study and test whether the benefits of hedging depend on scientists’ leadership and seniority.

## Concluding remarks

Existing research on knowledge networks has yet to answer how agentic responses to the opportunities afforded by networks shape scientific knowledge production. This paper is aligned to the increasing but scarce literature which characterizes the effects of networks from a behavioral perspective, as distinct (although complementary) to the perspective of conceptualizing these effects as a result of network structures. In the context of scientific knowledge production, our study advances our understanding of how network behaviors matter, by demonstrating how a precise network utilization decision – the practice of network hedging – helps the scientist to reduce the uncertainty inherent in scientific work and to produce high-impact science. We hope future research will develop this insight by zooming in on how specific network mobilization choices affect the individual ability to most effectively leverage social networks and isolate the effects on science and innovation. This would add to a finer-grained theoretical understanding of how network utilization decisions influence information processing and interpretation.

## Supporting information

S1 Section Response rate and usable responses by medical specialty. (DOCX)

S2 Section Survey items on scientists’ networks. (DOCX)

S3 Section Correlogram. (DOCX)

S4 Section Results for negative binomial regression. Explanatory variable: Average number of contacts per benefit.
(DOCX)

S5 Section Results for logistic regression. Alternative dependent variable: Ptop 10% as a dummy (Percentile 75 ≥ 1, otherwise = 0).
(DOCX)

S6 Section Results for negative binomial regression. Full model removing biomedical scientists with two or fewer network contacts.
(DOCX)

S7 Section Results for negative binomial regression. Winsorization of hedging values.
(DOCX)

S8 Section Results for the two sample Mann-Whitney test (N = 771). (DOCX)

S9 Section Results for OLS regression. Reverse causality. Dependent variable: Network hedging t-2.
(DOCX)

S10 Section Results for negative binomial regression. Exploring non-linear effects of network hedging on high scientific impact.
(DOCX)

S11 Section Results for negative binomial regression. Interplay between hedging and network diversity.
(DOCX)

S12 Section Results for negative binomial regression. Split sample analysis based on cognitive disparity. Dependent variable: *P*_*top10%*_.
(DOCX)
