# Supplementary material for: Negotiating knowledge: The role of network hedging in the production of high-impact science
Source: PLoS One. 2026 Jun 29;21(6):e0352349. doi: 10.1371/journal.pone.0352349 (PMC13313354; doi:10.1371/journal.pone.0352349)
Supplement: S1 Section — (DOCX) [file pone.0352349.s001.docx]

**SUPPLEMENTAL MATERIAL for**

**“Negotiating knowledge: The role of network hedging in the production of high-impact science”**

**Section S1.** Response rate and usable responses by medical specialty.

| **Medical specialty** | **Population surveyed (1)** | **Returned surveys (2)** | **Response rates for returned surveys (1) / (2)** | **Usable responses (3)** | **Response rates for usable responses (3) / (1)** |
| --- | --- | --- | --- | --- | --- |
| Bioengineering, biomaterials and nanomedicine | 872 | 238 | 27.30% | 133 | 15.25% |
| Diabetes and associated metabolic diseases | 331 | 96 | 29.00% | 63 | 19.03% |
| Liver and digestive diseases | 459 | 154 | 33.60%^*^ | 101 | 22.00% |
| Rare diseases | 517 | 177 | 34.20%^*^ | 115 | 22.24% |
| Respiratory diseases | 439 | 159 | 36.20%^*^ | 95 | 21.64% |
| Epidemiology and public health | 610 | 107 | 17.50%^*^ | 54 | 8.85% |
| Neurodegenerative diseases | 750 | 186 | 24.80% | 102 | 13.60% |
| Physiopathology of obesity and nutrition | 303 | 71 | 23.40% | 36 | 11.88% |
| Mental health | 477 | 121 | 25.40% | 72 | 15.09% |
| Total | 4,758 | 1,309 | 27.50% | 771 | 16.20% |

*Notes*: ^*^indicates significant statistical difference in response rates (p < 0.05). Statistical significance was calculated by comparing the relative frequency with which the surveyed scientists are classified into the categories of non-respondents and respondents (using a Chi-square test).
