# Supplementary material for: Negotiating knowledge: The role of network hedging in the production of high-impact science
Source: PLoS One. 2026 Jun 29;21(6):e0352349. doi: 10.1371/journal.pone.0352349 (PMC13313354; doi:10.1371/journal.pone.0352349)
Supplement: S2 Section — (DOCX) [file pone.0352349.s002.docx]

**Section S2**. Survey items on scientists’ networks.

*Network size.* Name generator question to capture respondents’ network of contacts beyond the regular research team: “Please, write down the names of those persons (up to ten) from outside your research lab who are particularly important for the advancement of your research activities.”

Subsequent questions activated with size depending on the number of alters reported:

*Network brokerage*. “Indicate if, according to your knowledge, the persons you have cited exchange information or advice with each other, in connection with their professional activities.” Symmetric matrix with each alter-alter possible tie.

*Network diversity*. “Indicate the sector or professional field of the persons cited as being a particularly important source of information or advice for your research activities.” Drop-down menu with the following options: basic researcher, applied researcher, medical practitioner, member of a patient association, industry, public administration, other.

*Network hedging*. Adapted from Cross and Sproull (2004) and Levin et al. (2011):

- *Problem reformulation*: help to define or reframe a problem.
- *Problem solving*: help with specific solutions to problems or technical advice.
- *Referral*: suggestions of other sources of information (people, archives, databases) not previously considered.
- *Validation*: validation of plans and solutions and bolstering confidence.
- *Credibility*: legitimation of ideas based on support from influential peers.

The provision of resources is not mutually exclusive for a given contact: the same contact can offer from one up to five different resources to the focal individual.
