# Supplementary figures and images for: Negotiating knowledge: The role of network hedging in the production of high-impact science
Source: PLoS One. 2026 Jun 29;21(6):e0352349. doi: 10.1371/journal.pone.0352349 (PMC13313354; doi:10.1371/journal.pone.0352349)

**Section S3**. Correlogram.


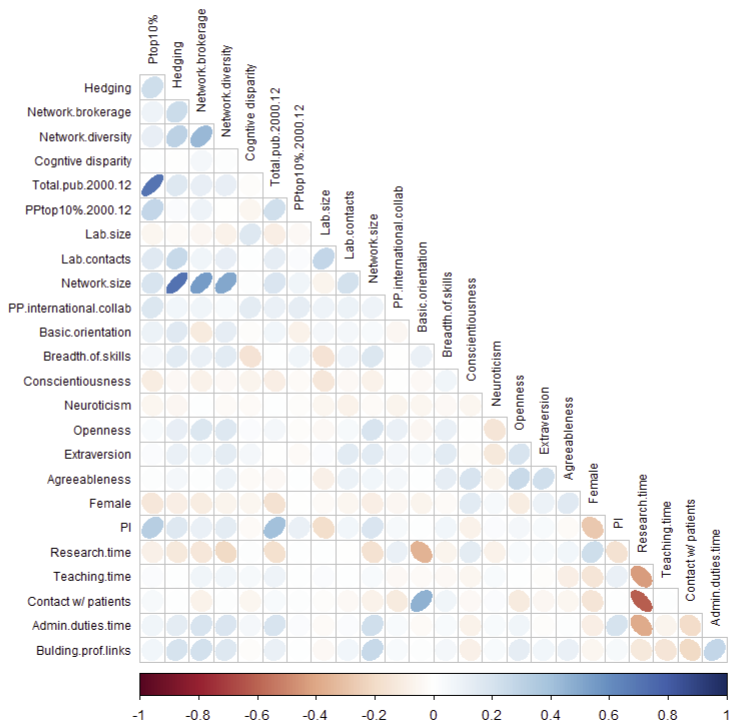


*Note*: Pearson correlations for all variables (N = 771).

Supplement: S3 Section — (DOCX) [file pone.0352349.s003.docx]
