# Supplementary material for: Negotiating knowledge: The role of network hedging in the production of high-impact science
Source: PLoS One. 2026 Jun 29;21(6):e0352349. doi: 10.1371/journal.pone.0352349 (PMC13313354; doi:10.1371/journal.pone.0352349)
Supplement: S4 Section — Explanatory variable: Average number of contacts per benefit. (DOCX) [file pone.0352349.s004.docx]

**Section S4.** Results for Negative Binomial Regression. Explanatory variable: Average number of contacts per benefit (N = 771).

|  | **M3: Hedging** | | **M4: Full model** | |
| --- | --- | --- | --- | --- |
|  | β (SE) | P-value | β (SE) | P-value |
| Hedging  (*average no. of contacts per benefit*) | 0.073 (0.030) | **0.015** | 0.089 (0.037) | **0.017** |
| Network diversity |  |  | 0.031 (0.060) | 0.606 |
| Network brokerage |  |  | 0.069 (0.027) | **0.011** |
| Cognitive disparity | 0.071 (0.038) | **0.060** | 0.068 (0.038) | **0.078** |
| Cognitive disparity sq | -0.120 (0.015) | **0.000** | -0.124 (0.014) | **0.000** |
| Total pub 2000–2012 | 0.537 (0.041) | **0.000** | 0.533 (0.047) | **0.000** |
| PP_top 10%_ 2000–2012 | 0.445 (0.089) | **0.000** | 0.447 (0.089) | **0.000** |
| Lab size | -0.007 (0.030) | 0.800 | -0.003 (0.027) | 0.907 |
| Lab contacts | 0.016 (0.037) | 0.671 | 0.014 (0.040) | 0.715 |
| Network size | 0.053 (0.060) | 0.374 | 0.001 (0.058) | 0.988 |
| PP_international collab._ | 0.237 (0.033) | **0.000** | 0.236 (0.031) | **0.000** |
| Basic orientation | -0.200 (0.074) | **0.007** | -0.201 (0.053) | **0.000** |
| Breadth of skills | 0.036 (0.027) | 0.182 | 0.032 (0.027) | 0.233 |
| Conscientiousness | 0.032 (0.057) | 0.581 | 0.032 (0.058) | 0.584 |
| Neuroticism | -0.041 (0.011) | **0.000** | -0.038 (0.014) | **0.005** |
| Openness | -0.020 (0.028) | 0.478 | -0.027 (0.030) | 0.369 |
| Extraversion | -0.001 (0.014) | 0.937 | -0.005 (0.012) | 0.696 |
| Agreeableness | -0.006 (0.021) | 0.764 | -0.007 (0.021) | 0.759 |
| Female | -0.082 (0.070) | 0.243 | -0.075 (0.055) | 0.174 |
| Principal investigator | 0.093 (0.054) | **0.085** | 0.090 (0.064) | 0.159 |
| University | -0.078 (0.018) | **0.000** | -0.091 (0.021) | **0.000** |
| Hospital | -0.063 (0.102) | 0.536 | -0.089 (0.092) | 0.334 |
| Public research org. | 0.063 (0.040) | 0.112 | 0.051 (0.042) | 0.224 |
| Research time | -0.038 (0.055) | 0.494 | -0.030 (0.054) | 0.581 |
| Teaching time | -0.033 (0.044) | 0.452 | -0.038 (0.046) | 0.404 |
| Contact w/ patients | 0.015 (0.086) | 0.861 | 0.018 (0.082) | 0.830 |
| Admin. duties time | -0.038 (0.031) | 0.216 | -0.043 (0.028) | 0.126 |
| Building prof. links | -0.008 (0.046) | 0.865 | -0.012 (0.043) | 0.775 |
| CIBER dummies | Yes |  | Yes |  |
| Constant | 1.511 (0.081) | **0.000** | 1.519 (0.074) | **0.000** |
| Cox & Snell *R*^2^ | 0.571 |  | 0.574 |  |

*Notes*: Robust standard errors are clustered by the type of institution affiliation of respondents and reported in parentheses. P-values in bold font indicate p < 0.10.
