# Supplementary material for: Negotiating knowledge: The role of network hedging in the production of high-impact science
Source: PLoS One. 2026 Jun 29;21(6):e0352349. doi: 10.1371/journal.pone.0352349 (PMC13313354; doi:10.1371/journal.pone.0352349)
Supplement: S5 Section — Alternative dependent variable: Ptop 10% as a dummy (Percentile 75 ≥ 1, otherwise = 0). (DOCX) [file pone.0352349.s005.docx]

**Section S5**. Results for Logistic Regression. Alternative dependent variable: Ptop 10% as a dummy (Percentile 75 ≥1, otherwise = 0 (N = 771).

|  | **Full model** | |
| --- | --- | --- |
|  | β (SE) | P-value |
| Hedging | 0.043 (0.016) | **0.006** |
| Network diversity | 0.061 (0.062) | 0.323 |
| Network brokerage | 0.114 (0.074) | 0.123 |
| Cognitive disparity | 0.112 (0.102) | 0.272 |
| Cognitive disparity sq | -0.262 (0.041) | **0.000** |
| Total pub 2000-2012 | 0.224 (0.049) | **0.000** |
| PP_top 10%_ 2000-2012 | 0.450 (0.065) | **0.000** |
| Lab size | -0.038 (0.038) | 0.323 |
| Lab contacts | -0.006 (0.014) | 0.649 |
| Network size | 0.044 (0.066) | 0.511 |
| PP_international collaboration_ | 0.173 (0.044) | **0.000** |
| Basic orientation | -0.235 (0.097) | **0.016** |
| Breadth of skills | 0.029 (0.068) | 0.668 |
| Conscientiousness | 0.043 (0.052) | 0.408 |
| Neuroticism | -0.003 (0.031) | 0.934 |
| Openness | -0.060 (0.037) | 0.103 |
| Extraversion | 0.015 (0.023) | 0.529 |
| Agreeableness | 0.021 (0.060) | 0.730 |
| Female | -0.151 (0.093) | 0.104 |
| Principal investigator | 0.112 (0.054) | **0.040** |
| University | -0.110 (0.131) | 0.399 |
| Hospital | -0.153 (0.207) | 0.459 |
| Public research org. | -0.012 (0.078) | 0.881 |
| Research time | 0.126 (0.156) | 0.421 |
| Teaching time | 0.112 (0.094) | 0.234 |
| Contact w/ patients | 0.142 (0.098) | 0.145 |
| Admin. duties time | 0.082 (0.084) | 0.332 |
| Building prof. links | 0.032 (0.031) | 0.293 |
| CIBER dummies | Yes |  |
| Constant | -0.973 (0.066) | **0.000** |
| Cox & Snell R^2^ | 0.183 |  |

*Notes*: Robust standard errors (SE) are clustered by the type of institution affiliation of respondents. P-values in bold font indicate p < 0.10.
