# Supplementary material for: Negotiating knowledge: The role of network hedging in the production of high-impact science
Source: PLoS One. 2026 Jun 29;21(6):e0352349. doi: 10.1371/journal.pone.0352349 (PMC13313354; doi:10.1371/journal.pone.0352349)
Supplement: S6 Section — Full model removing biomedical scientists with two or fewer network contacts. (DOCX) [file pone.0352349.s006.docx]

**Section S6**. Results for Negative Binomial Regression. Full model removing biomedical scientists with two or fewer network contacts (N = 505).

|  | **Full model** | |
| --- | --- | --- |
|  | β (SE) | P-value |
| Hedging | 0.057 (0.024) | **0.019** |
| Network diversity | 0.066 (0.038) | 0.079 |
| Network brokerage | 0.046 (0.032) | 0.150 |
| Cognitive disparity | -0.011 (0.023) | 0.643 |
| Cognitive disparity sq | -0.112 (0.029) | **0.000** |
| Total pub 2000-2012 | 0.469 (0.050) | **0.000** |
| PP_top 10%_ 2000-2012 | 0.478 (0.076) | **0.000** |
| Lab size | -0.027 (0.016) | 0.104 |
| Lab contacts | 0.050 (0.022) | 0.023 |
| Network size | 0.064 (0.085) | 0.451 |
| PP_international collab._ | 0.215 (0.056) | **0.000** |
| Basic orientation | -0.189 (0.055) | **0.001** |
| Breadth of skills | 0.018 (0.027) | 0.515 |
| Conscientiousness | -0.010 (0.067) | 0.880 |
| Neuroticism | -0.041 (0.035) | 0.244 |
| Openness | -0.026 (0.025) | 0.301 |
| Extraversion | -0.004 (0.019) | 0.821 |
| Agreeableness | 0.025 (0.018) | 0.158 |
| Female | -0.092 (0.049) | **0.060** |
| Principal investigator | 0.182 (0.086) | **0.034** |
| University | 0.101 (0.053) | **0.057** |
| Hospital | 0.049 (0.064) | 0.448 |
| Public research org. | 0.052 (0.038) | 0.174 |
| Research time | -0.073 (0.151) | 0.629 |
| Teaching time | -0.043 (0.078) | 0.577 |
| Contact w/ patients | 0.003 (0.125) | 0.984 |
| Admin. duties time | -0.066 (0.026) | **0.010** |
| Building prof. links | -0.005 (0.071) | 0.944 |
| CIBER dummies |  |  |
| Constant | 1.333 (0.170) | **0.000** |
| Cox & Snell R^2^ | 0.597 |  |

*Notes*: Robust standard errors (SE) are clustered by the type of institution affiliation of respondents. P-values in bold font indicate p < 0.10.
