# Supplementary material for: Negotiating knowledge: The role of network hedging in the production of high-impact science
Source: PLoS One. 2026 Jun 29;21(6):e0352349. doi: 10.1371/journal.pone.0352349 (PMC13313354; doi:10.1371/journal.pone.0352349)
Supplement: S7 Section — Winsorization of hedging values. (DOCX) [file pone.0352349.s007.docx]

**Section S7**. Results for Negative Binomial Regression. Winsorization of hedging values (N = 771).

|  | **Winsorization at top 5%** | | **Winsorization at top 10%** | |
| --- | --- | --- | --- | --- |
|  | **Full model** | | **Full model** | |
|  | β (SE) | P-value | β (SE) | P-value |
| Hedging | 0.056 (0.029) | **0.051** | 0.074 (0.010) | **0.000** |
| Network diversity | 0.068 (0.027) | **0.011** | 0.068 (0.027) | **0.014** |
| Network brokerage | 0.031 (0.063) | 0.620 | 0.03 (0.058) | 0.609 |
| Cognitive disparity | 0.067 (0.037) | **0.070** | 0.067 (0.038) | **0.073** |
| Cognitive disparity sq | -0.123 (0.013) | **0.000** | -0.124 (0.013) | **0.000** |
| Total pub 2000-2012 | 0.534 (0.045) | **0.000** | 0.533 (0.045) | **0.000** |
| PP_top 10%_ 2000-2012 | 0.446 (0.088) | **0.000** | 0.446 (0.087) | **0.000** |
| Lab size | -0.002 (0.026) | 0.937 | -0.002 (0.025) | 0.923 |
| Lab contacts | 0.018 (0.042) | 0.675 | 0.018 (0.043) | 0.675 |
| Network size | 0.044 (0.039) | 0.263 | 0.045 (0.046) | 0.323 |
| PP_international collab._ | 0.235 (0.031) | **0.000** | 0.235 (0.031) | **0.000** |
| Basic orientation | -0.207 (0.053) | **0.000** | -0.211 (0.050) | **0.000** |
| Breadth of skills | 0.032 (0.026) | 0.225 | 0.03 (0.026) | 0.244 |
| Conscientiousness | 0.032 (0.058) | 0.582 | 0.032 (0.058) | 0.585 |
| Neuroticism | -0.037 (0.015) | **0.014** | -0.038 (0.015) | **0.010** |
| Openness | -0.026 (0.030) | 0.386 | -0.027 (0.030) | 0.374 |
| Extraversion | -0.004 (0.013) | 0.788 | -0.004 (0.013) | 0.757 |
| Agreeableness | -0.008 (0.020) | 0.691 | -0.007 (0.020) | 0.722 |
| Female | -0.082 (0.052) | 0.116 | -0.083 (0.055) | 0.132 |
| Principal investigator | 0.099 (0.065) | 0.129 | 0.099 (0.067) | 0.140 |
| University | -0.092 (0.020) | **0.000** | -0.088 (0.021) | **0.000** |
| Hospital | -0.081 (0.096) | 0.401 | -0.077 (0.099) | 0.436 |
| Public research org. | 0.055 (0.039) | 0.161 | 0.059 (0.042) | 0.164 |
| Research time | -0.022 (0.046) | 0.624 | -0.022 (0.043) | 0.617 |
| Teaching time | -0.035 (0.043) | 0.420 | -0.035 (0.041) | 0.403 |
| Contact w/ patients | 0.024 (0.076) | 0.756 | 0.024 (0.074) | 0.750 |
| Admin. duties time | -0.04 (0.031) | 0.190 | -0.04 (0.030) | 0.185 |
| Building prof. links | -0.012 (0.043) | 0.774 | -0.012 (0.042) | 0.770 |
| CIBER dummies | Yes |  | Yes |  |
| Constant | 1.526 (0.074) | **0.000** | 1.532 (0.076) | **0.000** |
| Cox & Snell R^2^ | 0.573 |  | 0.573 |  |

*Notes*: Robust standard errors (SE) are clustered by the type of institution affiliation of respondents. P-values in bold font indicate p < 0.10.
