# Supplementary material for: Negotiating knowledge: The role of network hedging in the production of high-impact science
Source: PLoS One. 2026 Jun 29;21(6):e0352349. doi: 10.1371/journal.pone.0352349 (PMC13313354; doi:10.1371/journal.pone.0352349)
Supplement: S8 Section — (DOCX) [file pone.0352349.s008.docx]

**Section S8**. Results for the Two sample Mann-Whitney test (N = 771).

***H_0_***: there is no difference in hedging based on biomedical scientist location

***H_1_***: biomedical scientist located in Spain’s major cities have greater hedging values than those based elsewhere

| **Location** | **Observations** | **Rank sum** | **Expected** |
| --- | --- | --- | --- |
| Madrid or Barcelona | 476 | 187744.5 | 183736 |
| Elsewhere | 295 | 109861.5 | 113870 |
| Total | 771 | 297606 | 297606 |

z = -1.346

Prob > |z| = 0.178
