# Supplementary material for: Negotiating knowledge: The role of network hedging in the production of high-impact science
Source: PLoS One. 2026 Jun 29;21(6):e0352349. doi: 10.1371/journal.pone.0352349 (PMC13313354; doi:10.1371/journal.pone.0352349)
Supplement: S9 Section — Reverse causality. Dependent variable: Network hedging t-2. (DOCX) [file pone.0352349.s009.docx]

**Section S9**. Results for OLS Regression. Reverse causality. Dependent variable: Network hedging t-2 (n = 202).

|  | **Full model** | |
| --- | --- | --- |
|  | β (SE) | P-value |
| Ptop 10% | -0.029 (0.025) | 0.237 |
| Hedging t-1 | 0.377 (0.156) | **0.017** |
| Network brokerage | -0.032 (0.204) | 0.874 |
| Network diversity | 0.123 (0.167) | 0.463 |
| Cognitive disparity | 0.103 (0.279) | 0.713 |
| Cognitive disparity sq | 0.017 (0.185) | 0.926 |
| Total pub 2000-2012 | -0.063 (0.225) | 0.781 |
| PP_top 10%_ 2000-2012 | -0.121 (0.094) | 0.200 |
| Lab size | 0.042 (0.222) | 0.850 |
| Lab contacts | 0.169 (0.167) | 0.313 |
| Network size | 0.186 (0.338) | 0.583 |
| PP_international collab._ | 0.056 (0.185) | 0.764 |
| Basic orientation | -0.611 (0.465) | 0.191 |
| Breadth of skills | -0.037 (0.162) | 0.820 |
| Conscientiousness | 0.108 (0.165) | 0.512 |
| Neuroticism | 0.198 (0.158) | 0.210 |
| Openness | -0.146 (0.171) | 0.394 |
| Extraversion | 0.158 (0.165) | 0.339 |
| Agreeableness | 0.025 (0.178) | 0.889 |
| Female | -0.371 (0.356) | 0.298 |
| Principal investigator | 0.147 (0.456) | 0.747 |
| University | -0.017 (0.703) | 0.981 |
| Hospital | -0.045 (0.696) | 0.948 |
| Public research org. | 0.670 (0.679) | 0.325 |
| Research time | 0.253 (0.484) | 0.602 |
| Teaching time | 0.540 (0.300) | **0.074** |
| Contact w/ patients | 0.047 (0.426) | 0.913 |
| Admin. duties time | 0.046 (0.294) | 0.875 |
| Building prof. links | 0.227 (0.196) | 0.250 |
| CIBER dummies | Yes |  |
| Constant | 1.698 (0.888) | **0.058** |

*Notes*: Robust standard errors (SE) are clustered by the type of institution affiliation of respondents. P-values in bold font indicate p < 0.10.
