# Supplementary material for: Negotiating knowledge: The role of network hedging in the production of high-impact science
Source: PLoS One. 2026 Jun 29;21(6):e0352349. doi: 10.1371/journal.pone.0352349 (PMC13313354; doi:10.1371/journal.pone.0352349)
Supplement: S10 Section — Exploring non-linear effects of network hedging on high scientific impact. (DOCX) [file pone.0352349.s010.docx]

**Section S10**. Results for Negative Binomial Regression. Exploring non-linear effects of network hedging on high scientific impact (N = 771).

|  | **Full model** | |
| --- | --- | --- |
|  | β (SE) | P-value |
| Hedging | 0.009 (0.027) | 0.729 |
| Hedging sq | 0.012 (0.005) | **0.035** |
| Network brokerage | 0.033 (0.063) | 0.604 |
| Network diversity | 0.069 (0.026) | **0.008** |
| Cognitive disparity | 0.069 (0.036) | **0.060** |
| Cognitive disparity sq | -0.122 (0.013) | **0.000** |
| Total pub 2000-2012 | 0.534 (0.046) | **0.000** |
| PP_top 10%_ 2000-2012 | 0.448 (0.088) | **0.000** |
| Lab size | 0.002 (0.028) | 0.945 |
| Lab contacts | 0.017 (0.041) | 0.679 |
| Network size | 0.046 (0.039) | 0.235 |
| PP_international collab._ | 0.235 (0.029) | **0.000** |
| Basic orientation | -0.200 (0.050) | **0.000** |
| Breadth of skills | 0.038 (0.031) | 0.222 |
| Conscientiousness | 0.034 (0.057) | 0.549 |
| Neuroticism | -0.037 (0.017) | **0.026** |
| Openness | -0.024 (0.029) | 0.396 |
| Extraversion | -0.002 (0.014) | 0.914 |
| Agreeableness | -0.006 (0.018) | 0.756 |
| Female | -0.083 (0.052) | 0.106 |
| Principal investigator | 0.096 (0.066) | 0.145 |
| University | -0.092 (0.024) | **0.000** |
| Hospital | -0.085 (0.096) | 0.380 |
| Public research org. | 0.051 (0.041) | 0.220 |
| Research time | -0.029 (0.053) | 0.581 |
| Teaching time | -0.036 (0.044) | 0.406 |
| Contact w/ patients | 0.018 (0.082) | 0.823 |
| Admin. duties time | -0.041 (0.030) | 0.175 |
| Building prof. links | -0.011 (0.041) | 0.784 |
| CIBER dummies | Yes |  |
| Constant | 1.504 (0.077) | **0.000** |
| Cox & Snell R^2^ | 0.574 |  |

*Notes*: Robust standard errors (SE) are clustered by the type of institution affiliation of respondents. P-values in bold font indicate p < 0.10.
