# Supplementary material for: Negotiating knowledge: The role of network hedging in the production of high-impact science
Source: PLoS One. 2026 Jun 29;21(6):e0352349. doi: 10.1371/journal.pone.0352349 (PMC13313354; doi:10.1371/journal.pone.0352349)
Supplement: S11 Section — Interplay between hedging and network diversity. (DOCX) [file pone.0352349.s011.docx]

**Section S11**. Results for Negative Binomial Regression. Interplay between hedging and network diversity (N = 771).

|  | **Full model** | |
| --- | --- | --- |
|  | β (SE) | P-value |
| Hedging | 0.070 (0.016) | **0.000** |
| Network diversity | 0.070 (0.026) | **0.008** |
| Hedging*Network diversity | -0.010 (0.015) | 0.495 |
| Network brokerage | 0.034 (0.063) | 0.586 |
| Cognitive disparity | 0.068 (0.038) | **0.076** |
| Cognitive disparity sq | -0.123 (0.014) | **0.000** |
| Total pub 2000-2012 | 0.533 (0.046) | **0.000** |
| PP_top 10%_ 2000-2012 | 0.448 (0.088) | **0.000** |
| Lab size | 0.000 (0.027) | 0.994 |
| Lab contacts | 0.016 (0.040) | 0.697 |
| Network size | 0.023 (0.050) | 0.641 |
| PP_international collab._ | 0.235 (0.029) | **0.000** |
| Basic orientation | -0.198 (0.050) | **0.000** |
| Breadth of skills | 0.035 (0.029) | 0.226 |
| Conscientiousness | 0.032 (0.058) | 0.578 |
| Neuroticism | -0.036 (0.016) | **0.022** |
| Openness | -0.025 (0.030) | 0.410 |
| Extraversion | -0.003 (0.014) | 0.814 |
| Agreeableness | -0.007 (0.020) | 0.726 |
| Female | -0.078 (0.052) | 0.131 |
| Principal investigator | 0.094 (0.064) | 0.141 |
| University | -0.093 (0.022) | **0.000** |
| Hospital | -0.088 (0.095) | 0.353 |
| Public research org. | 0.049 (0.041) | 0.232 |
| Research time | -0.030 (0.053) | 0.571 |
| Teaching time | -0.038 (0.044) | 0.383 |
| Contact w/ patients | 0.019 (0.083) | 0.819 |
| Admin. duties time | -0.042 (0.029) | 0.148 |
| Building prof. links | -0.012 (0.042) | 0.769 |
| CIBER dummies | Yes |  |
| Constant | 1.519 (0.074) | **0.000** |
| Cox & Snell R^2^ | 0.574 |  |

*Notes*: Robust standard errors (SE) are clustered by the type of institution affiliation of respondents. P-values in bold font indicate p < 0.10.
