# Supplementary material for: Negotiating knowledge: The role of network hedging in the production of high-impact science
Source: PLoS One. 2026 Jun 29;21(6):e0352349. doi: 10.1371/journal.pone.0352349 (PMC13313354; doi:10.1371/journal.pone.0352349)
Supplement: S12 Section — Split sample analysis based on cognitive disparity. Dependent variable: Ptop10%. (DOCX) [file pone.0352349.s012.docx]

**Section S12**. Results for Negative Binomial Regression. Split sample analysis based on cognitive disparity. Dependent variable: *P_top10%_*

|  | **IQR = 1: Within interquartile range (n = 386)** | | **IQR = 0: Outside interquartile range (n = 385)** | |
| --- | --- | --- | --- | --- |
|  | **Full model** | | **Full model** | |
|  | β (SE) | P-value | β (SE) | P-value |
| Hedging | 0.012 (0.036) | 0.738 | 0.133 (0.062) | **0.031** |
| Network brokerage | 0.041 (0.042) | 0.325 | 0.021 (0.100) | 0.834 |
| Network diversity | 0.042 (0.044) | 0.340 | 0.123 (0.071) | **0.080** |
| Total pub 2000-2012 | 0.440 (0.047) | **0.000** | 0.792 (0.062) | **0.000** |
| PP_top 10%_ _2000-2012_ | 0.367 (0.051) | **0.000** | 0.515 (0.120) | **0.000** |
| Lab size | -0.057 (0.061) | 0.345 | -0.059 (0.036) | 0.106 |
| Lab contacts | 0.029 (0.029) | 0.307 | 0.010 (0.088) | 0.912 |
| Network size | 0.114 (0.083) | 0.167 | -0.071 (0.084) | 0.400 |
| PP_international collaboration_ | 0.226 (0.031) | **0.000** | 0.230 (0.038) | **0.000** |
| Basic orientation | 0.284 (0.091) | **0.002** | 0.109 (0.129) | 0.397 |
| Breadth of skills | 0.025 (0.034) | 0.464 | 0.055 (0.022) | **0.011** |
| Conscientiousness | 0.067 (0.067) | 0.324 | -0.041 (0.027) | 0.136 |
| Neuroticism | 0.011 (0.041) | 0.794 | -0.136 (0.053) | **0.011** |
| Openness | -0.048 (0.039) | 0.214 | 0.014 (0.049) | 0.778 |
| Extraversion | 0.005 (0.026) | 0.854 | -0.003 (0.057) | 0.957 |
| Agreeableness | 0.008 (0.045) | 0.862 | 0.005 (0.072) | 0.948 |
| Female | -0.126 (0.119) | 0.290 | -0.003 (0.054) | 0.959 |
| Principal investigator | 0.204 (0.053) | **0.000** | -0.219 (0.095) | **0.022** |
| University | 0.035 (0.064) | 0.584 | -0.262 (0.14) | **0.060** |
| Hospital | 0.019 (0.146) | 0.894 | -0.099 (0.139) | 0.476 |
| Public research org. | 0.071 (0.070) | 0.313 | 0.066 (0.099) | 0.502 |
| Research time | 0.186 (0.201) | 0.355 | -0.16 (0.227) | 0.481 |
| Teaching time | 0.069 (0.098) | 0.482 | -0.15 (0.127) | 0.239 |
| Contact w/ patients | 0.180 (0.166) | 0.276 | -0.14 (0.165) | 0.394 |
| Admin. duties time | 0.051 (0.087) | 0.560 | -0.097 (0.068) | 0.153 |
| Building prof. links | 0.011 (0.078) | 0.891 | -0.053 (0.113) | 0.637 |
| CIBER dummies | Yes |  | Yes |  |
| Constant | 0.831 (0.150) | **0.000** | 1.292 (0.293) | **0**.**000** |
| Cox & Snell R^2^ | 0.597 |  | 0.552 |  |

*Notes*: Robust standard errors (SE) are clustered by the type of institution affiliation of respondents. P-values in bold font indicate p < 0.10.
